# COVID-19: an opportunity to re-evaluate the implementation of a One Health approach to tackling emerging infections in Nigeria and other sub-Saharan African countries

**DOI:** 10.1186/s42506-021-00085-y

**Published:** 2021-08-20

**Authors:** Olaniyi Ayobami, Godwin Mark, Zaharat Kadri-Alabi, Chioma Rita Achi, Joy Chinwendu Jacob

**Affiliations:** 1 Department of Infectious Disease Epidemiology, Robert Koch Institute, Berlin, Germany; 2 Department of One Health, Royal (Dick) School of Veterinary Studies, The University of Edinburgh, Edinburgh, Scotland, UK; 3 Department of Internal Medicine, Federal Teaching Hospital Gombe, Gombe, Nigeria; 4 Department of One Health, Royal Veterinary College, London, UK; 5 Department of Veterinary Medicine, University of Cambridge, Cambridge, UK; 6 Department of Veterinary Public Health and Preventive Medicine, Usmanu Danfodiyo University Sokoto, Sokoto, Nigeria; 7 Department of Vaccinology Education, Universitat Autonoma de Barcelona, Barcelona, Spain

**Keywords:** Emerging infections, One Health, Nigeria, Sub-Saharan Africa

## Abstract

**Background:**

One Health (OH) has resurfaced in the light of the ravaging COVID-19 pandemic. It has been accepted by many local and global health authorities as a suitable approach for preventing and responding to infectious disease outbreaks including pandemics.

**Main body:**

One Health (OH) is a multisectoral and interdisciplinary framework for managing the animal, human, and ecosystem determinants of health. Globally, the majority of emerging infections in humans including SARS-Cov2—the causative agent of COVID-19—are transmitted from animals through environmental contacts in the last few decades. Yet, even when the biological and social interactions at the human, animal, and environmental interface that drive spillover of zoonotic diseases have been proven, OH strategies to address associated complex health challenges today are still rudimentary in many national health systems. Despite the disproportionate burden of infectious diseases in sub-Saharan Africa, OH is minimally incorporated into routine disease control and national health security programs. Challenges include poor policy support for OH in sub-Saharan Africa, and where some form of policy framework does exist, there are significant implementation bottlenecks. In this paper, we identified ideological, technical, operational, and economic barriers to OH implementation in Nigeria and sub-Saharan Africa, and highlighted possible recommendations across these domains. In order to yield sustainable benefits, a relevant OH policy approach in the sub-Saharan African health systems must derive from a buy-in of the critical mass of stakeholders in the society.

**Conclusion:**

The implementation of sustainable OH approaches as a countermeasure to recurring emerging infections is a developmental priority for sub-Saharan African countries. A deep understanding of the local context must be leveraged to develop integrative OH solutions that are bold, rooted in science, and proven to be compatible with the level of development in sub-Saharan Africa.

## Background

One Health (OH) is a multisectoral and interdisciplinary framework for managing the animal, human, and ecosystem determinants of health. There is increasing realization today that many emerging infections such as Ebola and COVID-19 is driven by the interaction of biological and social factors at the human, animal, and environmental interface [1].

After the outbreaks of severe acute respiratory syndrome (SARS) and the Middle East respiratory syndrome (MERS) in 2003 and 2012, respectively, efforts have been directed towards embedding OH within established global health institutions. However, health systems and emergency preparedness at the regional and national levels remain lack-lustre and faced with significant impediments. More than ever, the COVID-19 pandemic has laid bare the importance of OH considering its origin as an animal-human species spill over disease, and its rapid spread worldwide through travel and social mixing [2]. The OH nature of COVID-19 emergence has unsurprisingly led to the operationalization of some aspects of OH into the response programs of numerous countries to tackle the ongoing pandemic [3].

Traditionally, across many low-resource settings in sub-Saharan Africa (SSA), human and animal health sectors are fragmented and this reflects in the varying approaches of veterinary and medical sectors to zoonoses [4]. In the face of recurring outbreaks and pandemics driven by factors with cross-sectoral impacts, the status quo is no longer tenable. Before COVID-19, Rwanda is one of the very few countries in SSA with a functional national strategic plan for OH [5]. In Nigeria, apart from the integration of the National Veterinary Institute as a surveillance facility for COVID-19, there is an increasing number of veterinary public health professionals being engaged in the risk communication to the public [6]. Beyond these, however, the veterinary sector in Nigeria is still largely underutilized in the response to COVID-19 or infectious diseases in general. Scientific disciplines relevant to OH are not restricted to human and veterinary clinical specialties. It includes ecology, health economics and policy, anthropology, and artificial intelligence among others; all are important for sustainable control of large-scale infectious disease outbreaks in an interconnected world [7]. From an African healthcare system perspective, the biomedical laboratory scientist, considered a vital healthcare detective, uncovering and providing laboratory information from specimen analyses—which aids physicians in patient’s care remains underfunded, and suffers inadequate infrastructure necessary to support the quick resolution of disease outbreaks of COVID-19 scale across SSA [8].

Negating previously held assumptions, it has become increasingly clear that infectious diseases will hardly be relegated to history [9]. It has therefore become necessary to address the infectious diseases threats across boundaries using multi-disciplinary inputs from diverse sectors working locally and regionally to optimize the health of all species. The aim of this review is to discuss possible strategies for developing an OH policy approach for the SSA health systems, drawing from a critical analysis of the prevailing cross-cutting issues and implementation barriers.

### Current situation of One Health policy in selected sub-Saharan African countries

OH is gaining increasing recognition in the organization of health systems of some of the 46 sub-Saharan African States. However, the most exciting development in OH policy in SSA comes from Rwanda [5]. Rwanda has taken the lead toward a national strategic plan for OH in the region with a robust network of community health workers (CHWs): a practical approach to addressing the gap in human resources for health. Notably, Rwanda’s OH strategic plan recognizes a need for a cultural shift in the political and educational consciousness of Rwandans and clearly outlines modalities for grass-root engagement and gives ownership of the project to the people and professionals at various levels. However, interdisciplinary turf wars, expertise gaps, and infrastructural deficits remain worrisome challenges. Since Rwanda’s health and socio-political realities are similar to many SSA countries, it signals the potential challenges in the development and implementation of national OH action plans by other SSA nations.

Kenya in 2006 institutionalized a OH framework - aimed at readying emergency preparedness - which was deployed in 2006 in the response to the Highly Pathogenic Avian Influenza (HPAI) and 2006/7 for Rift Valley Fever (RVF) [10]. The success of this scheme led to the formation of the Zoonotic Diseases Unit (ZDU) in 2012 to enhance disease surveillance and support public health response [10]. The efforts culminated in the establishment of county OH units supported through the U.S Centers for Disease Control and Prevention (CDC), Kenya’s Global Health Security Agenda (GHSA) implementing partners [10]. How these initiatives have contributed to the COVID-19 response in Kenya remains to be assessed.

In 2008, the Nigeria Federal Ministry of Agriculture and Federal Ministry of Health developed a strategy that birthed the Nigeria Field Epidemiology and Laboratory Training Program (NFELTP) [11], with support from the CDC. This OH framework has aggregated the efforts of veterinarians, medics, and laboratory scientists in emergency response and tackling infectious diseases within the country; however, the depth of inclusion and operation of the environmental sector in this framework is still poor. Despite its active involvement in the implementation of awareness programs on the roles that hygiene, water, sanitation, and proper waste disposal play in curtailing the spread of pathogens and antimicrobial resistant bugs, the environmental health sector in Nigeria struggles with budgeting shortfalls, unstructured operating procedures, and inadequate integration into the prevention and response systems for emerging/re-emerging zoonoses. It is also less well invested in climate change, wildlife, and ecosystem preservation. Importantly, the National Action Plan on Antimicrobial Resistance (AMR) for 2017–2022 takes into consideration the tripartite structure of OH for AMR surveillance, although still in its early stages of implementation. Overall, there is increasing consciousness and comparison of notes among professionals culminating in a recent OH strategic plan vested with the Nigerian Centre for Disease Control (NCDC) [12].

Tanzania has pockets of OH programs mainly from numerous partnerships, academic programs, and research interventions. The Bill and Melinda Gates Foundation funds a rabies elimination programme targeted at the Serengeti wildlife ecosystems, Selous, and other regions [13]. The University of Glasgow provides the technical and research support for rabies interventions in Tanzania mainland and Zanzibar relying on a OH approach [14]. Also, through a novel OH approach, public health professionals, OH students, the Food and Agriculture Organization (FAO), and the government have formed partnerships for rabies control in the Moshi, Kilimanjaro region [15]. However, the challenge with project-based interventions remain their sustainability, national ownership, funding constraints, and lack of an overarching OH policy.

As of 2021, Ghana is yet to enact a OH policy although signatory to the Global Health Security Agenda (GHSA). Ghana nevertheless has a National Action Plan for Health security. Through that framework, the GHSA and Veterinary Services Directorate (VSD) have intensified efforts to strengthen the national surveillance laboratory systems for swift detection and response to prioritised zoonotic diseases. OH workforce capacity in Ghana is enhanced through the [16] Training Programmes in Epidemiology and Public Health Interventions Network (TEPHINET) in core areas like applied epidemiology, public health, and laboratory practice skills. Despite widespread support for the OH concept in Ghana, resource limitations, nonexistence of a clearly defined multisector operational framework, and inadequate collaboration among key governmental agencies impacts Ghana’s ability to effectively mitigate or respond to infectious biosecurity threats.

For the rest of SSA, whilst concrete policy frameworks are mostly lacking, there are a number of OH networks and programs scattered across the region. These initiatives are cataloged by the One Health Centre in Africa (www.ilri.org/research/facilities/one-health-centre). The identified weaknesses of such networks range from limited to absent information sharing culture among critical stakeholders, poor operational and coordinating mechanisms, minimal involvement of sectors other than public and veterinary health, paucity of clear implementation guidelines, disciplinary “tribalism” and territory protections, and a lack of institutionalization of OH concepts in the relevant organizations.

### Challenges of implementing One Health in sub-Saharan Africa

OH policy limitations in Nigeria and SSA are mainly ideological and operational. Barriers also exist at the tactical, political, economic, research, and strategic levels [17–20].

*Ideological* limits occur in the sense that definitions of OH and its scope are unclear to many potential stakeholders. Many human physicians for instance, who have heard about OH may not appreciate how they can marry traditional emphasis on the human aspects of disease with the animal or ecological determinants of health. Previous studies have pointed to the amorphous nature of the OH concept as a significant limitation to implementing the OH agenda in a globally agreeable and context-relevant manner [18, 20].

Next, there are *“operational”* barriers, in terms of the day-to-day application of OH to solve complex health challenges like the ongoing COVID-19 scourge. Across many SSA countries, limited information sharing and poorly delineated operational linkages among the health, environment, agriculture, and veterinary ministries underlie the tokenistic interactions commonly seen after a disease outbreak. For example, the Ethiopian public health and veterinary health authorities only began collaborating during the Reft Valley Fever (RVF) outbreak in 2006. In Nigeria, despite multiple waves of Lassa and other zoonotic diseases, the operationalization of the national OH strategic plan still has a negligible implementation score.

Although it is expected that OH in Africa operates within existing public health frameworks at this early stage, the nature of its integration will depend on the individual country capacity and circumstances like the level of existing intersectoral cooperation among other factors. The need for such strategic clarity is more acute in many SSA countries due to, pre-existing poverty, and developmental challenges. Aside from these cross-cutting challenges, each OH discipline has peculiar operational challenges that must be considered into the design of the OH framework. For instance, the emergence of COVID-19, and before that, of SARS and H5N1 Avian influenza from wet markets in Asia, indicates that wet animal markets are hotspots for emerging and re-emerging infectious diseases [21]. At wet markets, humans, wild, and domestic animals are often brought together near one another, often under undue stress, presenting an opportunity for efficient sharing of pathogens and the emergence of novel ones [22]. These features characterize many animal-trade markets in Nigeria and many African countries. Closing wet markets in Nigeria may increase exposure to animal pathogens, as markets, including slaughterhouses, are known to grow organically even within residential areas.

At the *‘tactical’* or *‘technical’* level, the limited diagnostic capacity among research and public health laboratories in Africa is exacerbated by inadequate networking and data exchange within and across OH sectors. Despite the scale-up of molecular testing for COVID-19 in Africa, test rates are still insufficient and generally fall short of the massive testing required for many well-populated African countries [23, 24]. Still, less than 1% of Nigeria’s 200 million and 0.5% of South Africa’s 60 million population has been tested despite the very high degree of social mixing and population mobility in these countries [25, 26]. The emergency scale-up of laboratory capacity seen in many SSA countries is not a sustainable national strategy to counter the threat of emerging infections. Without a strategy to facilitate the integration of overlapping input factors (knowledge, methods, training, and communication), it might remain difficult to effectively operationalize OH on a wide scale. The Canadian model is a veritable example of OH in action, through the adoption of the same building for veterinary and human diagnostics and sharing of databases and information resources while ensuring the highest level of biocontainment. The model has incontrovertibly demonstrated the promotion of shared value and resource efficiency [27].

From a *“policy/political”* perspective, sectoral barriers that divide public health, from veterinary, environmental, food safety policies, and other sectors of OH activities are common. To date, there are still limited opportunities for medical doctors, vets, environmentalists, and farmers to exchange information and brainstorm at the local level. Developing robust policy frameworks must go beyond identifying the need for OH, to determining the necessary level of cooperation and concerns for preserving the integrity of individual disciplines. Also, should the formal and informal expertise merge? These are complex questions that need to be answered as the push for the creation and adoption of a relevant OH framework compatible with the level of development in Africa intensifies.

From an *“economic”* standpoint, there are funding constraints for implementing OH in SSA. The current COVID-19 pandemic has paralyzed the global economy and is tipped to result in the severest economic recession seen in history [28]. It is argued, that, the reliance on global aid, would no longer prove sustainable in the coming months should COVID-19 persist. SSA needs innovative economic approaches to save costs by maximizing existing resources to drive the OH agenda.

### Recommendations

At the *ideological* level, we propose prioritization of national strategic OH plans that deepens the inclusion of environmental health as a key element of the OH framework. It is on such a premise that the animal and environmental health practice—hitherto unappreciated—can be rejuvenated and strengthened to foster understanding and drive the much-needed paradigm change. Such transdisciplinary partnerships should translate into benefits that are beyond merely additive consultations. At the *policy level*, it is necessary to push for “plueridisciplinary” educational, training, and research across all the disciplines that make up the OH ecosystem. It is high time that the desire to break professional silos in OH driven by genuine efforts to foster collaboration among stakeholders in a bottom-up approach. Nigeria and other sub-Saharan African states can take the lead in deepening OH integration in education, training, and research by encouraging students and professionals to share classroom and field practices where feasible. Such integration will help to reframe communication about emerging infections, biosecurity risks across sectors, and financing in health policy debates using approaches simple enough for the policymakers and the society at large to understand.

At the *operational* level, various health agencies will need to explore opportunities for linkages and information sharing on wildlife, livestock, and human diseases. Identified priority OH interventions and strategies must be underpinned by an evidence base that is locally determined, employing appropriate integration mechanisms to enhance interoperability among the disparate OH agencies and ministries [29].

At the *technical* level, priority interventions should focus on boosting the human resources for health, including the enhancement of the diagnostic capacity for infectious diseases sustainably. For instance, since veterinarians and animal health professionals have an incentive to work in communities, empowering them will provide value like animal disease surveillance information, and risky behaviors communication to animal owners. The frequent interactions of rural dwellers with wildlife further strengthen the case for zoonotic disease management at the local level. Due to its transnational dimension, a repository rich in essential cross-country health, ecological, and animal migration and trade data will also enhance regional biosecurity through early warning.

From a *political* and *economic* viewpoint, the added value of OH in terms of outbreak prevention, containment, and cost-savings needs to be continuously demonstrated to policymakers through economic analysis and research. This need becomes acute when we admit that the quicker sustainable solutions are devised for recurring epidemics in Africa, the earlier the region can divert scarce resources to other important development and economic recovery initiatives. To upturn dependence on foreign support, an initial step could be setting up a dedicated “national OH fund” drawing from the annual budgets of ministries of health, agriculture and environment, and other non-governmental sources. Special funding initiatives like the Nigerian Basic Healthcare Provision Fund can be repurposed to accommodate the increasing need for OH interventions.

## Conclusion

One of the most important barriers of OH as a national policy is how to redirect stakeholder priorities from the traditional human-centric concentration to a holistic paradigm that esteems these three pillars of OH equally. COVID-19 has laid bare yet again the importance of the OH and provides an opportunity to strengthen strategies for public health and infectious disease management in SSA. Tackling the COVID-19 pandemic, and indeed mitigating the next outbreak, calls for a One Health approach - an approach that integrates existing resources, research, and intervention efforts among human, animal, and environmental health sectors in a manner compatible with the level of development. A contextually relevant OH strategic plan for Nigeria and SSA is one that is robust enough to deal with the current implementation barriers across multiple levels of the health governance. Future research should explore the “how” and the extent to which cross-disciplinary collaboration should be pursued ethically, and how to mobilize societal support and position OH at the front burner of health policy discourse.

## Data Availability

Not applicable

## Abbreviations

AMR: Antimicrobial resistance
FAO: Food and Agriculture Organization
OH: One Health
SARS: Severe acute respiratory syndrome
MERS: Middle East respiratory syndrome
SSA: Sub-Saharan Africa
CHWs: Community health workers
HPAI: Highly Pathogenic Avian Influenza
RVF: Rift Valley fever
ZDU: Zoonotic Diseases Unit
CDC: U.S Center for Disease Control and Prevention
GHSA: Global Health Security Agenda
NFELTP: Nigeria Field Epidemiology and Laboratory Training Program
NCDC: Nigerian Centre for Disease Control
VSD: Veterinary Services Directorate
